# American Association of Medical Editors

**Published:** 1870-07

**Authors:** S. M. Bemiss

**Affiliations:** Acting Secretary; Washington, D.C.


### AMERICAN ASSOCIATION OF MEDICAL EDITORS.

WASHINGTON, D.C., *May 2, 1870.*

Pursuant to adjournment, the "Association of American Medical Editors" met in the lecture room of Georgetown Medical College, at 10 o'clock A.M., on Monday, May 2d, 1870.

The Permanent Secretary and Secretary being absent, Dr. Bemiss stated that Dr. Mitchell had requested him to supply his place, if absent, but had not placed the books containing the minutes and correspondence in his hands. At the President's instance, Dr. Bemiss took the Permanent Secretary's place, and read from a daily paper the proceedings of the last meeting. The minutes, as read, were corrected by the addition of Dr. Parvin's name, as one of the signers of the Articles of Association.

Dr. Yandell moved that the name of Dr. Charles A. Lee, of New York, be added, on account of long and distinguished services as a journalist. Carried unanimously.

Dr. Parvin, as Chairman of the Committee on Foreign Exchanges, reported verbally that he had found great difficulty in effecting arrangements for exchange with the editors of foreign journals, and suggested that editors resident upon the seaboard possessed advantages in such matters over those living as far inland as himself.

On motion of Dr. Bemiss, Dr. Parvin was continued, with power to add the names of such editors as he might select to aid him.

On motion of Dr. Parvin, Dr. Antisell was elected a member of this Association.

The Permanent Secretary failed to report in regard to instructions given him to correspond with Editors of Journals, informing them of the objects of this Association, and requesting their co-operation.

The Committee on Registering of Names of Physicians failed to report. The Committee on Revision failed to report. The Committee to arrange a general plan of communication between Medical Journals failed to report.

The names of Dr. John A. Murphy, of the Cincinnati *Lancet and Observer*, and of Drs. H. Carpenter and J. C. Grubbs, of the Oregon *Medical and Surgical Reporter*, were added to the list of those signing the Articles of Association.

On motion of Dr. Parvin, it was agreed to adjourn until 8½ P.M., to meet in this hall, and hear the President's address upon the history, progress, etc., of Medical Journalism.

HALL OF GEORGETOWN MEDICAL COLLEGE, *May 2, 8 P.M.*

The Association met pursuant to adjournment. The names of Dr. W. T. Briggs, of the Nashville *Journal of Medicine and Surgery;* Dr. Horatio R. Storer, of the *Journal* of the Gynæ-logical Society of Boston; of Dr. E. Lloyd Howard, of the *Baltimore Medical Journal;* Dr. Ed. Warren, of *Medical Bulletin;* and Dr. B. F. Dawson, of the *American Journal of Obstetrics*, were added to those subscribing to the Articles of Association.

The Committee on Registry was continued to the next annual meeting.

The Association then proceeded to the annual Election of Officers.

On nomination by Dr. Parvin, Dr. H. R. Storer, of Boston, was elected President.

On nomination by Dr. Bemiss, Dr. Theophilus Parvin was elected Vice-President, and Dr. Howard, of Baltimore, Secretary. The President then read his address.

ADDRESS ON THE HISTORY, CONDITION, AND MEANS OF IMPROVEMENT OF MEDICAL JOURNALISM IN THE UNITED STATES. BY N. S. DAVIS, M.D., PRESIDENT OF THE ASSOCIATION.

*Gentlemen of the Association:*—In strict obedience to the directions given me by the Association, at its organization in New Orleans, on May 5th, 1869, I rise to address you on this occasion. That the medical periodical press is at the present time one of the most important means of eliciting and diffusing medical knowledge, and consequently aiding in the education of the profession, but few thoughtful men will be inclined to deny. The first medical periodical published in America, of which we have any knowledge, was the *Medical Repository*, commenced in 1797, in the city of New York, and edited by Drs. Sam'l L. Mitchell, Edward Miller, and Elihu H. Smith.

It was a good sized quarterly journal, and its pages were enriched by the contributions of many of the ablest members of the profession at that date. Dr. Mitchell remained its principal editor through the first sixteen volumes, when it passed under the editorial management of Dr. James R. Manley, who, with his associates, maintained its reputation and usefulness until the end of the twenty-third volume.

In September, 1804, the *Medical Museum* was commenced in Philadelphia, edited by Dr. John Redman Coxe. In the same city, in November of the same year, the first number of the *Medical and Physical Journal* was issued, under the editorial management of Dr. Benj. Smith Barton.

The *Museum* was continued until 1813, while we lose all trace of the *Medical and Physical Journal* in 1808, until it is revived under the same name, and edited by Nathaniel Chapman, in 1820, and continued until 1827, when it seems to have been merged into the *American Journal of Medical Sciences*, which has continued until the present time. Another quarterly journal, called the *American Medical Recorder*, was commenced in Philadelphia, in 1818, and continued until 1829, when it appears to have been also merged into the *American Journal of Medical Sciences.*

A neatly illustrated quarterly journal, devoted to Medicine, Natural History, and Agriculture, called the *American Medical and Philosophical Register*, was published in New York, in 1810, and continued until 1814. The New England *Journal of Medicine, Surgery, and Collateral Branches*, was published in Boston, from 1812 to 1824. After 1820, new medical periodicals continued to be issued so rapidly, that I have been wholly unable to devote sufficient time to the subject to enable me to follow them in detail. With the aid of Dr. Toner, of Washington, I have been able to learn the names of about one hundred and twenty medical periodicals proper, which have been issued within the last fifty years, not including the annual transactions of Medical Societies.

Of these, one-half were discontinued within from six months to three years from the commencement of their publication. Of the remaining number, twenty did not continue beyond five years; and of more than thirty medical periodicals belonging legitimately to the profession, not including those of dentistry, now being published in the United States, only thirteen have been published more than a single decade.

With its present form and title, the *American Journal of Medical Sciences*, quarterly, has been published with commendable regularity since 1827, a period of forty-three years; and if it were proper to regard it as only a new name and form for the *Philadelphia Journal of Medical and Physical Sciences*, its origin could be dated back, under Dr. Chapman, to 1820, or even to 1804, under Dr. Benj. Smith Barton.

In 1823, Dr. J. V. C. Smith commenced the publication of the *Medical Intelligencer*, in Boston, which appears to have been changed to the *Boston Medical and Surgical Journal*, in 1827, and under that title, continued until the present time. It has been issued in both weekly and monthly form, to suit the wishes of its subscribers. Of the regular monthly and bi-monthly Journals, now in course of publication, the *Lancet and Observer*, at Cincinnati, dates its commencement, under the title of the *Western Lancet*, in 1841; the *St. Louis Medical and Surgical Journal*, in 1843; the *Chicago Medical Journal* and the *New Orleans Medical and Surgical Journal*, in 1844; and the *Buffalo Medical and Surgical Journal*, in 1845.

If this mere glance at the history of Medical Journalism in our country shows that a very large proportion of our periodicals have been unstable and evanescent, a similar inquiry in reference to those who have been connected with them in the capacity of editors, will show a still greater degree of instability. For instance, of the thirteen medical periodicals now existing, which were represented to have been published continuously more than a single decade, not more than seven have been under the same editorial management, during that short period of time. At the present writing, we count thirty-three medical periodicals belonging properly to the profession, in the United States. It is proper to state that we do not include in the list, Journals devoted especially to dentistry, the annual publications of Medical Societies, nor certain sheets designed as the advertising mediums of wholesale drug manufacturers. Of the thirty-three included in the list, six are published quarterly, twenty-two monthly, one semi-monthly, and four weekly. Four of the quarterlies and one of the monthlies are devoted to the cultivation of special departments, namely, the *American Journal of Insanity*, published at Utica, N. Y; the *Journal of*

*Psycological Medicine*, published at New York; the *American Journal of Obstetrics and Diseases of Women and Children*, and the *American Journal of Sypholography and Dermatology*, in the same city, are issued quarterly, and in good style. The *Journal of the Gynæcological Society*, of Boston, devoted to the Diseases of Women, is published monthly at Boston.

The two remaining quarterlies, the *American Journal of Medical Sciences*, at Philadelphia, and the *New Orleans Journal of Medicine*, with the twenty-two monthlies, one semi-monthly, and the four weeklies, are devoted to general medicine.

It is thus seen, that notwithstanding the numerous abortive attempts at Medical Journalism which has characterized our past history, we still have an abundant medical periodical literature, the publication of which is well distributed to the various parts of our country. In regard to the present character of our medical periodicals, I am constrained to speak with some hesitation. Twenty-two years of active, editorial labor in connection with the medical press, has certainly afforded me opportunity to become familiar with the subject.

I cannot agree with those, who, after excepting one or two favorite publications, unsparingly denounce all the rest as mere trash, or stupid copyists from one another. Neither can I join with those, at home or abroad, who indulge in unrestricted depreciative comparison of our periodicals with those of other countries.

And yet there are faults too obvious to be overlooked, and which this Association should make earnest efforts to correct. Nearly all our periodicals are modeled on the same plan. Each contains a department for original articles, and clinical and Society reports; another for selections from other periodicals; another for notices of new publications, and another for editorial and miscellaneous matter.

It is true that most of our periodicals admit into the first of these departments articles defective in style, and impoverished in ideas; cases so imperfectly reported as to be of no value; and reports from Hospitals and Societies, equally imperfect and valueless, except to fill space, and give publicity to the names of the parties concerned. But a candid examination will show that matter of this character really occupies but a small part of the space devoted to original matter, in nine-tenths of our Medical Journals. In the department for selected matter, there is room for great improvement in a large proportion of our Journals. In some, it habitually occupies from half to two-thirds of the whole number of pages, and consequently gives the publication more the character of a reprint than anything else. In some cases, this is done by copying in full articles from other Journals, and in others, by making summaries or digests of the current matter in their exchanges.

The occasional copying of an essay or article of more than ordinary value, from one journal to another, always giving due credit for its source, and thereby giving it a wider circulation, is doubtless not only justifiable, but beneficial to the author and the whole profession. But as a general rule, the editors of medical journals should treat the original matter in their cotemporaries in the same manner as new publications. They should make such careful and well considered reviews of the important papers as would give their readers a correct idea of their nature and value, thereby enabling them to judge whether the original was worth their purchase or not. In a large proportion of our journals, the space devoted to new books has come to be filled with a simple announcement of the work, with a copy of the title-page, and sometimes the table of contents. The quarterlies, however, and a few of the monthlies, present in each number some very creditable reviews.

But, in our estimation, no part of our medical periodicals is more deficient than that which is expected to be filled by editorial matter proper. Some of them contain so little from the editorial pen, that their readers would hardly know that they were superintended by such a functionary, except as they see his name on the title page. Others present one or two pages headed "editorial," and filled with facetious paragraphs, personal slurs, news items, and attention to new advertisements. Only a very few out of the whole number occupy what editorial space they have with candid articles, calculated to enlighten their readers on the many important questions connected with the sanitary, social, ethical, and educational interests of the profession. And I think it may be said with truth, that in none of our periodicals do these topics receive the editorial attention that their importance demands.

It is only through the periodical press that the great body of the profession can be reached. To sit in an editorial chair and occasionally growl at municipal authorities for neglecting sanitary measures; slur medical societies for imperfections in their action; indite facetious paragraphs about ethics; and ridicule the attempts of others to advance educational interests, is, to say the least, a very poor performance of the duties belonging to such a position.

Before leaving this branch of our subject, we feel constrained to allude to two other items which we have long regarded as detrimental to the character of a large part of our medical periodicals. One consists in a large amount of editorial puffing and pretension about the superior quality of their particular periodical, and the extraordinary arrangements they have just completed for making it the *very best* in the country.

The rule of ethics, founded on a just sense of propriety, forbids an ordinary practitioner from boasting of his success in practice, or of the extraordinary character of his remedies. And we cannot see why it is not equally in bad taste for the editor or publisher of a medical journal to indulge in a similar strain of bombast about his own work.

The other relates to the kind and number of advertisements that are appended to each issue. To the insertion of a list of new medical books, new surgical instruments and appliances, or the simple business card of a physician, druggist, or medical institution, we do not object. But to take up a periodical, professedly devoted to the interests of science and a learned profession, and find one-third or one-half of its entire bulk made up of advertisements of all kinds, from Fougera's pills to wooden tubs and sewing machines, is equivalent to receiving the impression that the publisher, having embarked in the advertising business, had employed an editor to furnish just enough professional matter to serve the same purpose that the tail does to a kite.

Having trespassed so much on your time and patience already, I will hasten to a brief consideration of the question, how can medical journalism in our country be improved? To answer this question satisfactorily, involves a correct appreciation of the causes of its past and present instability and imperfection. These have been very generally attributed to the excessive number published, and the consequent inadequate pecuniary return for the labor required. And the remark is often made, that if our medical periodicals were restricted in number to such as are issued from a few of the great centres of population, where their editors have access to the abundant materials afforded by Hospitals and Medical Societies, they would command a sufficient number of readers and supporters to give them permanence and excellence. It is very doubtful whether this view can be verified, either by observed facts, or the application of well established mental laws.

If it were true that the discontinuance of one-half of the medical periodicals now in the course of publication would result in transferring all the patronage they have received to the remaining half; or if the patronage bestowed on each new journal was simply so much taken from the journals previously existing, then, indeed, would the number published furnish a direct index of the amount of patronage each could expect to receive. But observation has long since satisfied us that the matter is governed by no such simple rule. On the contrary, every new journal started enlists a circle of friends and patrons, a large part of whom were taking no periodical before, and contributing to none.

And if it so happens that the circle formed is too restricted and the enterprise fails, it will have created in many of its patrons the habit of reading and writing, which will remain and be turned to the benefit of some other periodical. It is a law of the human mind that the more its faculties are exercised, either in the acquisition or communication of knowledge, the more imperious are its demands for an additional supply. The practitioner who has read or contributed to one medical journal, is far more likely to add another to the list, than he is to discontinue the first. A full examination will show that the unstable and imperfect character of many of our medical periodicals is neither owing to their number, nor to the fact that the places of their publication are widely diffused over our country. There are practitioners enough in the United States, if each subscribed for only one periodical, to afford every medical journal, now in course of publication, a fair support.

And the publication of these periodicals, in the different and distant sections of our country, is of great advantage in developing a knowledge of the climate, topography, and diseases of their respective regions, as well as stimulating a taste for reading and writing in the local profession around them. Our experience and observations have satisfied us that most of the faults connected with American medical journalism are traceable to two sources, namely, the defective education of the profession, and the imperfect arrangements of those who undertake the editorial supervision and publication of the respective journals.

It is perfectly well known that a large part of those who enter upon the practice of medicine, under our system of medical education, are wholly destitute of that general education and mental discipline which is essential to the formation of a taste for reading and writing, Without an adequate knowledge of the elementary branches of common education, and without the slightest acquaintance with any of the sciences, they have performed the task of reading the text-books in medicine, much as the apprentice performs his task in a mechanic's shop; they have attended the heterogeneous courses of Lectures, in some Medical College, during which they have made imperfect notes of as many formulas or prescriptions for particular diseases as they could, and they enter upon practice with full confidence that these formulas and their text-books furnish all the literature necessary for the rest of their lives. They have no taste for reading, and not the slightest appreciation of the value of medical periodicals.

And if here and there one of this class is induced to patronize a journal, or furnish a contribution, the letter is written in such style, that the editor must either throw it into his basket of waste paper, so far re-write it, that the author would not recognize it as his, or let it appear in such condition as to disgrace the pages of his journal. It is directly to this imperfect education of the profession, that medical journalism owes both its limited patronage and the literary imperfections which have so frequently subjected it to disparaging criticism. The physician whose mind has been early disciplined by study, and fed with the bread of science, will be just as much lost without one or more medical periodicals, as is the clergyman without his church paper, or the politician without his party organ.

The second efficient cause of instability in medical journals was stated to be, the imperfect arrangements of those who undertake their editorial supervision and publication. The history of a large proportion of them may be briefly stated as follows:

The Faculty of a College wants an *organ;* or one or two young men, laudably ambitious, think that an editorial position would both give them notoriety and access to the current medical literature; and in either case, a bookseller, or publisher, or some other business firm, who can be made to think that the proposed journal would be a profitable medium for advertising his own wares, and that enough additional advertisements can be obtained to pay a large part of the expense of publication, is sought out, a bargain made, a prospectus issued, soon followed by the first number of the work. The members of the college faculty whose names have been put on as editors, or the ambitious young men whose names occupy that position, have provided no reliable corps of reporters to furnish what can be gleaned from the Hospitals and Medical Societies, if any such exist in the neighborhood; they have no resources for original matter, except the voluntary contributions of members of the profession, and what is equally bad, they have no positive views of medical polity, medical education, or sanitary science, with which to give their own editorial space a positiveness and individuality calculated to attract attention and command respect.

By personal solicitation, they succeed in obtaining contributions enough from their friends to make a respectable show of original matter for the first few numbers, but this resource is soon exhausted, and they are obliged to increase their selections from other journals, to fill up the required number of pages; the fear of offending some interest, whose patronage is needed, deters from doing more in the editorial department than to write commendatory notices of books, or call attention to some new advertisement, until heartily tired of the enterprise, they discover that they have not *sufficient time* to devote to the work, and either let it die, or induce some new man to undertake it, and go through the same process.

In other instances, where the editor holds out more tenaciously, notwithstanding his scanty supply of material, the publisher, after one or two years, discovers that the benefit he derives from the advertising medium is not equal to the deficiency of receipts as compared with the expenditures, and he withdraws, leaving another printer to be found, who, in turn, arrives at the same conclusion in a few months. If the foregoing views are correct, in regard to the causes of the insufficient patronage, instability and imperfections of medical periodicals, in this country, the remedies are obvious. Nothing short of a higher standard of education, both preliminary and medical, on the part of those who enter the profession and a more correct appreciation of the arrangements and qualifications required for maintaining a creditable medical journal, will remedy the evils. The first would multiply the number of readers and ensure the proper merit in their contributions, while the second would speedily arrest the tendency to make inconsiderate efforts to establish new journals.

Towards the accomplishment of these purposes, this Association has it in its power to contribute much. Prominent among the declared objects of this Association, as set forth in the act of its organization, are the cultivation of friendly relations and mutual assistance, *concert* of action in support of improvements in the present system of medical education, and of a higher standard of preliminary attainments for those who propose to enter upon the study of medicine, and in promoting generally the value and efficiency of our periodical medical literature.

If all those who occupy the responsible position of editors of medical periodicals in this country, will co-operate, by becoming members of this organization, and prove true to its principles and purposes, it can wield a more direct and powerful influence for good, both in elevating the character of American medical journalism, and in increasing the honor and usefulness of the whole profession, than any other organization in existence. You who control the medical press hold in your hands the main avenues through which the great mass of the professional mind can be reached and influenced. You have the means and the power, if you choose to use them, to mould the public sentiment of the profession and concentrate it on the accomplishment of any desirable object, with an irresistable force.

Dr. Theophilus Parvin, at whose suggestion this Association was formed last year, stated, as one of the reasons for such action, that the editors of the medical periodical press were not exerting that positive influence on the medical public which belonged to their position, simply because a large part of them maintained a studied silence on all the important topics to which we have alluded, while others break their silence only by an occasional facetious remark. Is it not time, gentlemen, that this apathy, this studied silence, on topics of so much importance, was abandoned? Is its continuance compatible with a just appreciation of the importance of our position and of our individual responsibility? If we have assumed positions that give us the power to wield an important influence for good, are we not justly responsible for the enlightened and efficient exercise of that power? These are questions that must be answered to our own consciences.

Then, let us perpetuate and extend our organization. Let each member honestly cultivate friendly relations with every other. Let us take concerted action, by out-spoken, candid, full discussion of the vital questions involved in the elevation of the standard of medical education, until the great evils universally acknowledged to exist are removed, and the profession in our country rests on an educational basis commensurate with the extent of its science and the nobleness of its art. But in all our work, let us remember that personalities are not arguments; that to pull down a rival is not equivalent to building oneself up; and that it takes far less time to inflict a wound than to heal it. Finally, my brethren of the editorial fraternity, let us justly appreciate both the influence and the responsibility which attaches to the position we occupy, and with honest, earnest purpose wield the one and respond to the other, in such a manner as will advance the true interests of our profession, because, in so doing, we shall most efficiently promote the interests of humanity.

On motion of Dr. Storer, the President was voted the thanks of the Association for his address, and its publication was requested, in any mode which he regarded as most practicable and politic.

The President announced that he would print the whole of these proceedings, including the address, and send advance sheets to the various medical journals of the country.

Dr. N. S. Davis was appointed Committee on Revision, to report at the next meeting.

On motion of Dr. Bemiss, Dr. J. M. Toner, of D.C., was requested to read, at the next annual meeting, a paper upon the "Curiosities of the Literature of American Medical Journals."

On motion of Dr. Bowling, it was agreed by the Editors present that each should furnish Dr. Toner his Journal for the coming year, free of charge, that he might be enabled to carry the wishes of the Association into effect, without too great presonal expenditure.

On motion, adjourned to meet on the Monday preceding the meeting of the American Medical Association for 1871, and at the same place.

S. M. BEMISS, *Acting Secretary.*

